# The ESD Robustness and Protection Technology of P-GaN HEMT

**DOI:** 10.3390/mi16111269

**Published:** 2025-11-11

**Authors:** Yijun Shi, Yantao Chen, Liang He, Xinghuan Chen, Yuan Chen, Guoguang Lu

**Affiliations:** The Science and Technology on Reliability Physics and Application of Electronic Component Laboratory, China Electronic, Product Reliability and Environmental Testing Research Institute, Guangzhou 511370, China; syj20094870@sina.com (Y.S.); aa243422722@126.com (Y.C.); xhchen01@outlook.com (X.C.); luguog@126.com (G.L.)

**Keywords:** P-GaN HEMT, electrostatic discharge (ESD), ESD protection technology, transmission line pulse (TLP), trigger voltage

## Abstract

This work first analyzes the failure behaviors of P-GaN HEMTs with different gate structures (Schottky gate vs. Ohmic gate) under both forward and reverse ESD stresses. It reveals that the Schottky gate structure lacks effective electrostatic charge discharge paths, which leads to the accumulation of transient charges generated by ESD stress in the gate terminal, resulting in significant transient overvoltage and ultimately causing breakdown failure. Subsequently, the paper systematically reviews three existing unidirectional ESD protection technologies based on the P-GaN HEMT platform. While these technologies can discharge transient electrostatic charges generated by both forward and reverse ESD stresses, they operate in diode mode during reverse ESD events, exhibiting excessively low reverse triggering voltage. Furthermore, unidirectional ESD protection structures based on resistive voltage division and diode voltage division introduce substantial forward and reverse leakage currents. Finally, the article evaluates four bidirectional GaN ESD protection technologies. These bidirectional structures can likewise discharge transient charges from both forward and reverse ESD stresses. Compared to unidirectional approaches, the key advantage of bidirectional ESD protection lies in its ability to provide an appropriate reverse triggering voltage during reverse ESD events, thereby effectively clamping the reverse potential to the desired level. However, likewise, bidirectional ESD protection schemes based on resistive or diode voltage division also inevitably introduce relatively large forward and reverse leakage currents.

## 1. Introduction

Gallium nitride (GaN) high electron mobility transistors (HEMTs) have garnered increasing attention in power electronics applications due to their significant advantages including high critical breakdown electric field, high electron saturation drift velocity, excellent high-temperature operating capability, and low specific on-resistance [1,2,3,4,5,6]. However, in practical applications, the gate structure of the enhancement-mode (*E*-mode) Schottky-gate P-GaN HEMTs exhibits vulnerability to transient high-speed electrical stress events, such as Electrostatic Discharge (ESD) event. Their gate structure functions as two reversely series-connected diodes, lacking effective electrostatic charge discharge paths. This results in electrostatic charge accumulation within the gate terminal during ESD stress, generating substantial transient overvoltage that ultimately damages the gate structure. Concurrent research reveals that the reverse Human Body Model (HBM) failure voltage (*V*_HBM_) of Schottky-gate P-GaN HEMTs does not exceed 0.5 kV, significantly below the industry benchmark of 2 kV [2,3]. Enhancing the ESD reliability of Schottky-gate P-GaN HEMT’s gate structure remains a critical research priority.

For lateral power devices such as Schottky-gate P-GaN HEMTs, monolithic integration of protection/control circuits with power devices is relatively straightforward, making lateral ESD protection technologies highly significant [7,8,9,10,11]. Zhou et al. [12], Chen et al. [13,14], and Shi et al. [15,16] proposed three distinct planar unidirectional ESD protection schemes capable of simultaneously discharging transient electrostatic charges generated by both forward and reverse ESD stresses. Nevertheless, during reverse ESD events, these structures operate in diode conduction mode with critically low reverse triggering voltage (*V*_trig_). Furthermore, unidirectional ESD protection architectures based on resistive/diode voltage division introduce substantial forward and reverse leakage currents. Certain applications require negative gate bias to ensure complete channel turn-off and prevent false triggering [17]. Consequently, the ESD protection technology with bidirectional clamping capability is required to clamp the gate potential to the desired voltage value during both forward and reverse ESD stress events while avoiding affecting the normal operation of the device. To address this, Shi et al. [18,19,20] and Ma et al. [21] developed multiple lateral bidirectional ESD protection technologies, all providing effective bidirectional ESD protection through simultaneous transient charge discharge. Unlike unidirectional counterparts, bidirectional technologies maintain appropriate reverse triggering voltages during reverse ESD events to clamp gate potentials at desired levels. Similarly, bidirectional implementations utilizing resistive/diode voltage division still introduce relatively large forward/reverse leakage currents.

This work first analyzes the failure behaviors of P-GaN HEMTs with different gate structures under both forward/reverse ESD stresses. Subsequently, it summarizes existing lateral unidirectional and bidirectional ESD protection technologies implemented on the P-GaN HEMT platform while evaluating their respective merits and limitations.

## 2. ESD Evaluation Models and Evaluation Methods

### 2.1. Electrostatic Discharge Models

Electrostatic discharge models can generally be categorized into three types: the Human Body Model (HBM), the Machine Model (MM), and the Charged Device Model (CDM).

#### 2.1.1. Human Body Model (HBM)

**Figure 1:**
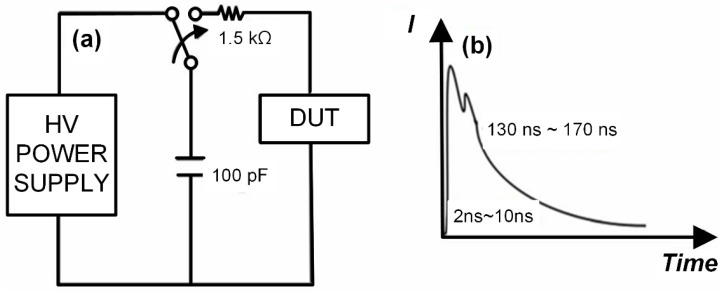
Human Body Model: (**a**) Equivalent Circuit Diagram; (**b**) Discharge Waveform Diagram.

This model simulates the scenario in which a charged human body discharges static electricity to an electronic device. In daily activities, electrostatic charge can accumulate on the human body due to friction, such as from clothing or floor contact. When a person comes into contact with the pins of an electronic device, the electrostatic charge carried by the body can transfer to the internal components. Figure 1 illustrates the equivalent circuit diagram and discharge waveform of the human body model. Typically, the human body capacitance is defined as approximately 100 pF, and the human body resistance is about 1.5 kΩ. During discharge, the electrostatic charge carried by the human body is released to the device through this equivalent circuit model. The discharge process is characterized by a relatively short duration, often lasting from several hundred nanoseconds to a few microseconds, with a typical decay time constant in the range of 150–200 ns. Despite the short time scale, the energy delivered can be substantial, leading to peak current levels reaching several amperes within a few hundred nanoseconds. Such high transient currents can induce severe electrical overstress, resulting in physical damage to semiconductor devices—including junction breakdown, metallization burnout, or gate oxide failure—and ultimately cause permanent device malfunction. This risk is especially critical for sensitive integrated circuits such as CMOS technologies, where the gate oxide layer is highly vulnerable to high-voltage transients. Even at electrostatic potentials as low as a few thousand volts, an HBM-type discharge can easily exceed the dielectric strength of thin oxide layers, leading to ruptures and functional failure. Therefore, the HBM test standard is widely adopted in the industry to evaluate the robustness of electronic components against ESD events originating from human handling.

#### 2.1.2. Machine Model (MM)

**Figure 2:**
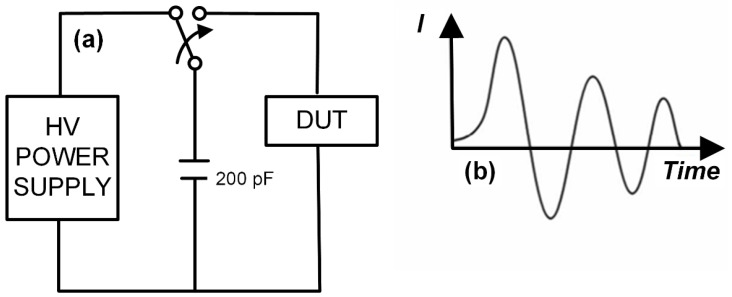
Machine Model: (**a**) Equivalent Circuit Diagram; (**b**) Discharge Waveform Diagram.

Metallic equipment (such as robotic arms in automated production lines, machine housings, etc.) or workstations may accumulate electrostatic charges due to friction, induction, or other reasons during operation. Without proper grounding, these accumulated electrostatic charges are difficult to dissipate. In the integrated circuit industry and the manufacturing process of semiconductor power devices, it is almost impossible to avoid direct contact between metallic equipment or workstations and integrated circuit chips or semiconductor power devices. As is well known, the charges inherently tend to transfer from high potential to low potential to achieve charge balance. This leads to the accumulation of charges on or inside integrated circuit chips and semiconductor power devices, a process classified as the Machine Model of electrostatic discharge. Since metallic equipment is a good conductor, the capacitance in the Machine Model is relatively small, typically around 200 pF, while the resistance is very low, usually less than 10 Ω. This results in a faster rise time for the discharge current, and a concentrated release of discharge energy within a short period. Figure 2 shows the equivalent circuit diagram and discharge waveform of the Machine Model. It can be observed that the Machine Model is characterized by a very short discharge time and a high peak current, delivering a more intense impact to the device. Such rapid and high-energy discharges can cause severe consequences, such as fusing of internal metal interconnects or damage to semiconductor structures. For example, during chip manufacturing, if automated equipment carries electrostatic charges, a discharge via the Machine Model can instantly damage the delicate circuit structures on the chip.

#### 2.1.3. Charged Device Model (CDM)

**Figure 3:**
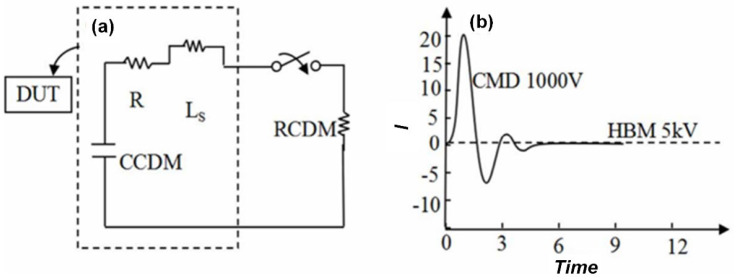
Charged Device Model: (**a**) Equivalent Circuit Diagram; (**b**) Discharge Waveform Diagram.

This refers to the phenomenon where during processes such as production, transportation, and assembly, devices may become charged with electrostatic electricity due to factors like friction or induction. When the device pins come into contact with a grounded object, the charge is rapidly discharged. If the devices lack efficient conductive pathways internally, the charge distributes uniformly within the device. At this stage, the device maintains overall charge balance, so no damage occurs. However, when the device carrying a significant amount of charge comes into contact with a good conductor, a conductive path is formed almost instantaneously. The uniformly distributed charge within the device rapidly discharges through the pins, creating a discharge pathway and resulting in a Charged Device Model discharge event. As shown in Figure 3, which illustrates the Charged Device Model, during discharge, the charge forms a discharge loop through the parasitic capacitance and resistance between the device pins and the grounded conductor. Compared to the Human Body Model, the Charged Device Model discharge duration is much shorter, typically on the nanosecond scale, with a very high peak current that can reach tens of amperes or even higher. This rapid and high-current discharge can cause severe damage to the internal circuits and structures of the device, such as destroying metal interconnects. The discharge mode of the Charged Device Model is influenced by the device’s own parameters. The capacitance and resistance of the device itself vary depending on its structure and materials. The discharge amount and current within the short duration are proportional to the unit area of the device and the accumulated charge.

The three electrostatic discharge models described above are the most commonly studied in research and exhibit considerable stability in experimental and production environments. Consequently, test results derived from these models show a high degree of consistency with actual electrostatic discharge phenomena observed in semiconductor devices. In practical production applications, the manufacturing and transportation of power devices inevitably involve human handling. Since humans are highly prone to carrying electrostatic charges, in-depth investigation of the Human Body Model electrostatic discharge mechanism is both critically important and urgent. As a result, the industry has adopted electrostatic discharge events triggered by the Human Body Model as the sole benchmark for electrostatic discharge testing.

### 2.2. Transmission Line Pulse (TLP) Testing Platform

**Figure 4:**
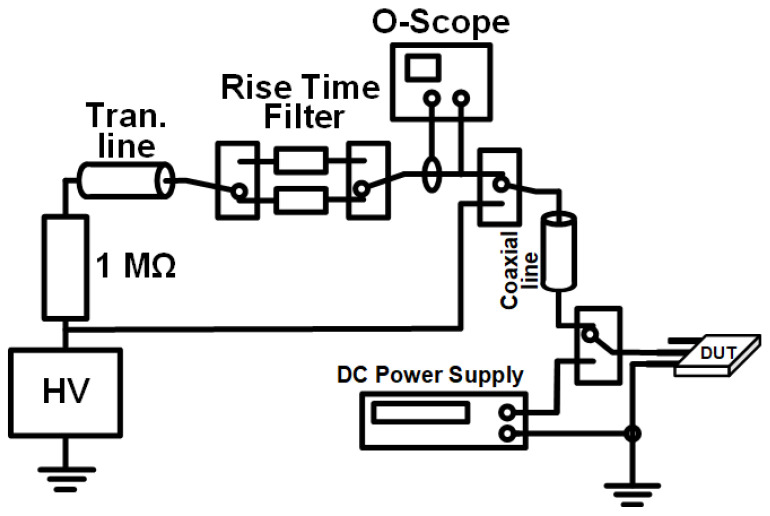
Schematic Diagram of the Transmission Line Pulse Testing System.

The Transmission Line Pulse test involves applying an escalating narrow pulse to the semiconductor device, simulating electrostatic discharge events. Extensive experimental data in the scientific community has confirmed the consistency between TLP testing results and the Human Body Model. The TLP test is widely adopted for analyzing the ESD robustness of electronic components. All ESD experimental results presented in this paper were obtained using a TLP measurement system. Figure 4 illustrates the schematic diagram of a Transmission Line Pulse testing system, which utilizes the characteristics of transmission lines to generate high-voltage pulses with specific waveforms and parameters. Its core operating mechanism involves charging a coaxial transmission line using a high-voltage DC power supply and then rapidly releasing the electromagnetic energy stored in the line to the device under test (DUT) via a high-speed switch. This generates a high-power pulse across the DUT with a duration equal to twice the electrical length of the transmission line and an extremely fast rise time. The pulse rise time is a key performance parameter of the TLP system, as it directly determines the highest frequency component that can be simulated and the accuracy of the test results. It is typically controlled by optimizing the switch characteristics and incorporating high-frequency filters to suppress unwanted resonance and ringing, thereby ensuring the purity and repeatability of the pulse waveform. The pulse duration is another critical performance parameter of the TLP system, determined by the electrical length of the transmission line.

## 3. ESD Robustness of P-GaN HEMT’s Gate Structure

**Figure 5:**
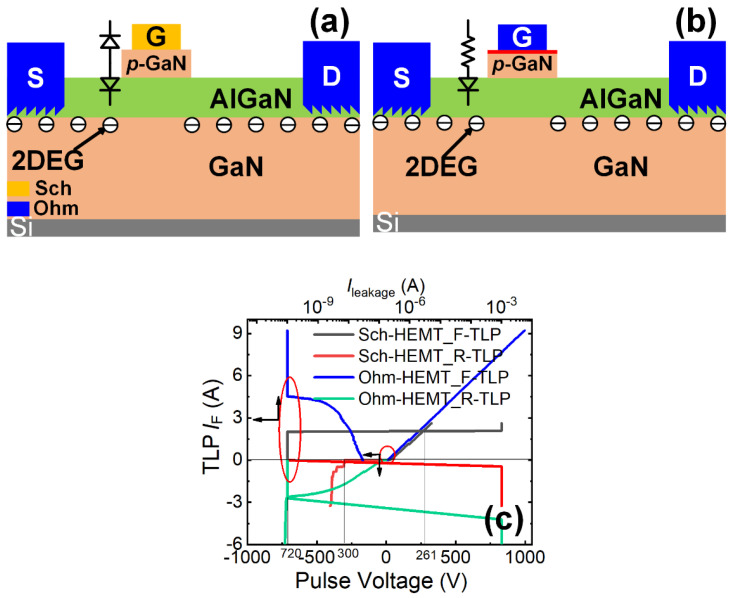
(**a**) Device structure of the Schottky-gate P-GaN HEMT; (**b**) Device structure of the Ohmic-gate P-GaN HEMT; (**c**) Bidirectional TLP *I-V* characteristics of Schottky-gate (Sch1/Sch2) and Ohmic-gate (Ohm1/Ohm2) P-GaN HEMTs, where Sch1 (Ohm1) and Sch2 (Ohm2) are subjected to forward and reverse gate-to-source (G-to-S) ESD stress, respectively [22].

Initially, we conducted a comparative analysis of forward/reverse gate ESD robustness between a Schottky-gate (Sch) P-GaN HEMT from GaN Systems (GS6511-1L (From Infineon Inc., Munich, Germany), with a rated voltage/current of 650 V/15 A) and an Ohmic-gate (Ohm) P-GaN HEMT from Infineon (IGLD60R190D1 (From Infineon Inc., Munich, Germany), with a rated voltage/current of 600 V/12.8 A), whose simplified device schematic structures are illustrated in Figure 5a,b. Unlike the Schottky-gate P-GaN HEMT, the Ohmic-gate variant features a thin heavily doped p-type GaN layer grown atop the P-GaN layer in the gate region. This enables Ohmic contact formation between the gate metal and the heavily doped P-type GaN layer, thus facilitating efficient hole injection from the P-GaN layer into the two-dimensional electron gas (2DEG) channel under high gate voltage conditions.

**Figure 6:**
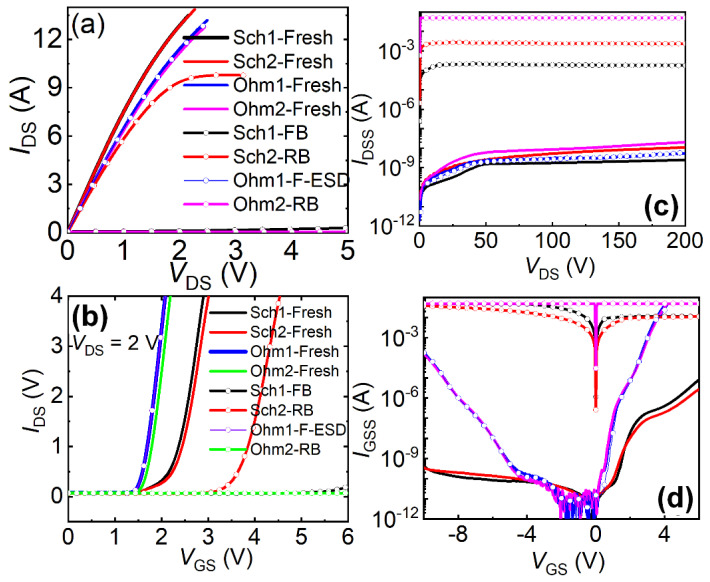
Output characteristics (**a**), transfer characteristics (**b**), drain leakage current (**c**), and gate leakage current (**d**) of the Schottky-gate and Ohmic-gate P-GaN HEMTs before and after forward ESD breakdown (FB)/reverse ESD breakdown (RB) [22].

**Figure 7:**
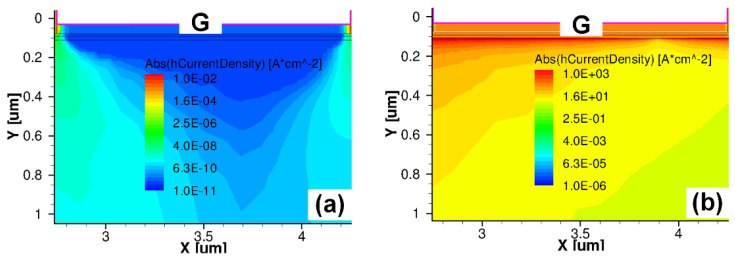
Simulated hole current density of the (**a**) Schottky-gate P-GaN structure and (**b**) Ohmic-gate P-GaN structure [22].

Figure 5c presents the forward/reverse gate-to-source (G-to-S) TLP I-V characteristics of Schottky-gate and Ohmic-gate P-GaN HEMTs [22,23]. Under the maximum applied forward pulse voltage (1000 V), the Ohmic-gate P-GaN HEMTs exhibited no forward ESD breakdown, with post-stress electrical characteristics (output/transfer/leakage properties) showing negligible degradation (Figure 6a–d). In contrast, the Schottky-gate device suffered breakdown under the applied pulse voltage at 261 V, demonstrating severe deterioration in output/transfer/leakage characteristics post-breakdown (Figure 6a–d). The forward breakdown current of the Ohmic-gate P-GaN HEMTs exceeded 9.3 A, significantly surpassing that of the Schottky-gate P-GaN HEMTs (2.0 A). The calculated equivalent forward Human Body Model failure voltages (*V*_HBM-F_ = 1500 Ω × breakdown current) were 14 kV (Ohmic-gate) and 3 kV (Schottky-gate). The superior forward ESD robustness of the Ohmic-gate P-GaN HEMT originates from the efficient hole injection from the P-GaN region into the 2DEG channel: TCAD simulations (Figure 7a,b) confirm sustained high hole current density of the Ohmic-gate P-GaN HEMT even at lower TLP voltages, indicating rapid transient charge dissipation through this pathway. This hole transport mechanism aligns with the thermionic emission theory [24,25,26], and its gate leakage current characteristics satisfy the equation:

(1) $$LN\left(I_{GSS}\right)\sim 2LN(T)$$

**Figure 8:**
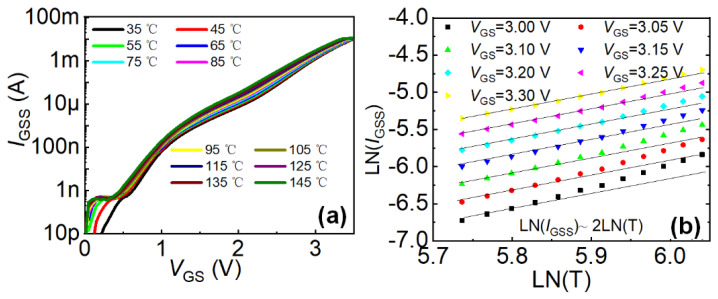
(**a**) Effect of temperature on the gate leakage current of Ohmic-gate P-GaN HEMTs, (**b**) ln(*I*_GSS_) vs ln(*T*) at different gate-to-source voltages, where the slope of the solid line is 2 [22].

LN is the notation for taking the logarithm with base “e”. To verify that the gate leakage current in Ohmic-gate P-GaN HEMT under high gate bias is primarily dominated by hole current, the temperature dependence of the gate leakage current was systematically investigated (Figure 8a,b). Experimental results demonstrate that the gate leakage current of Ohmic-gate P-GaN HEMT increases significantly with rising temperature and strictly follows the relationship described by Equation (1), confirming that the hole transport dominates the gate leakage current under high gate bias conditions. Conversely, in Schottky-gate P-GaN HEMT, the reverse Schottky barrier at the metal/P-GaN interface severely impedes hole injection into the 2DEG channel, resulting in inadequate transient charge dissipation capability and consequently inferior forward G-to-S ESD robustness.

The reverse G-to-S TLP characteristics in Figure 5c reveal a higher reverse breakdown current in Ohmic-gate P-GaN HEMTs (2.7 A) compared to Schottky-gate devices (0.1 A). Correspondingly, the reverse equivalent HBM failure voltages were 4.05 kV (Ohmic-gate) and 0.15 kV (Schottky-gate). Both the Ohmic-gate and Schottky-gate P-GaN HEMTs exhibited substantial electrical degradation after reverse ESD breakdown (Figure 6a–d). The enhanced reverse robustness of Ohmic-gate devices stems from their unique charge dissipation pathway—efficient reverse hole injection from the 2DEG channel to the Ohmic gate electrode. In contrast, Schottky-gate structures suffer from dual charge blocking mechanisms: the Schottky barrier at the metal/P-GaN interface forces hole accumulation at the interface, while the AlGaN/GaN heterojunction barrier impedes electron injection from the gate metal to the 2DEG channel. This absence of effective charge dissipation channels critically compromises their reverse G-to-S ESD robustness.

## 4. ESD Protection Technology for P-GaN HEMT’s Gate Structure

### 4.1. Unidirectional ESD Protection Technology

#### 4.1.1. Resistive Voltage-Division and Diode Voltage-Division Unidirectional ESD Protection Technologies [12,13,14,16,20]

**Figure 9:**
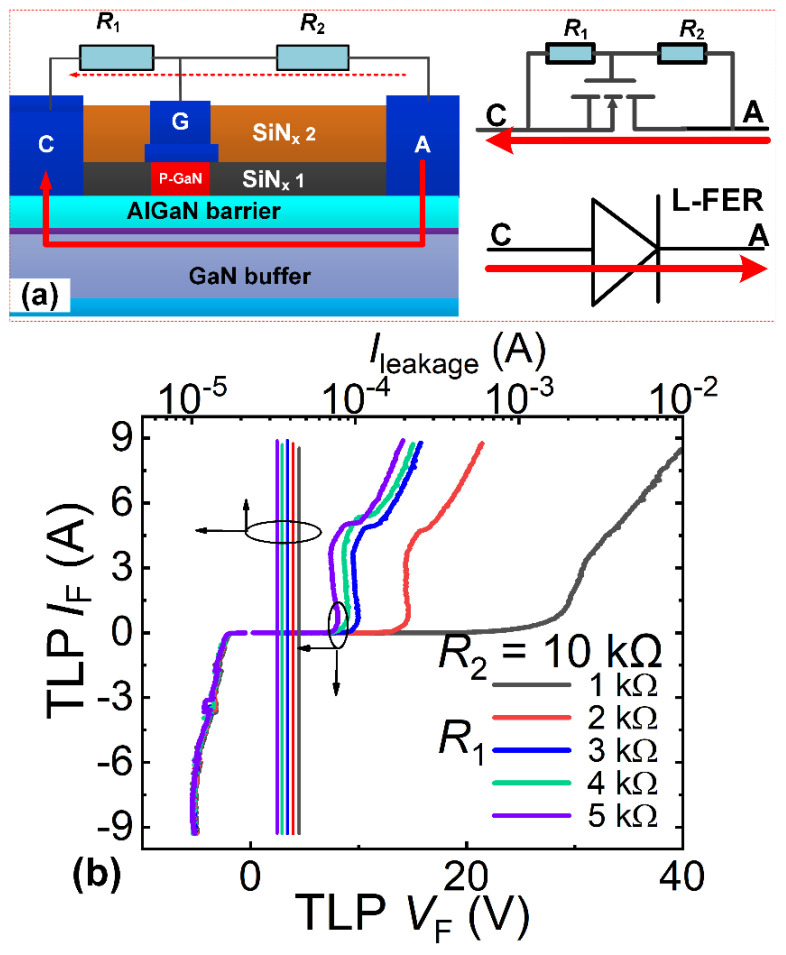
(**a**) Schematic diagram of the resistive voltage-division unidirectional ESD protection structure and its bidirectional conduction model; (**b**) Bidirectional TLP I-V characteristics of the resistive voltage-division unidirectional ESD protection structure [12,16].

**Figure 10:**
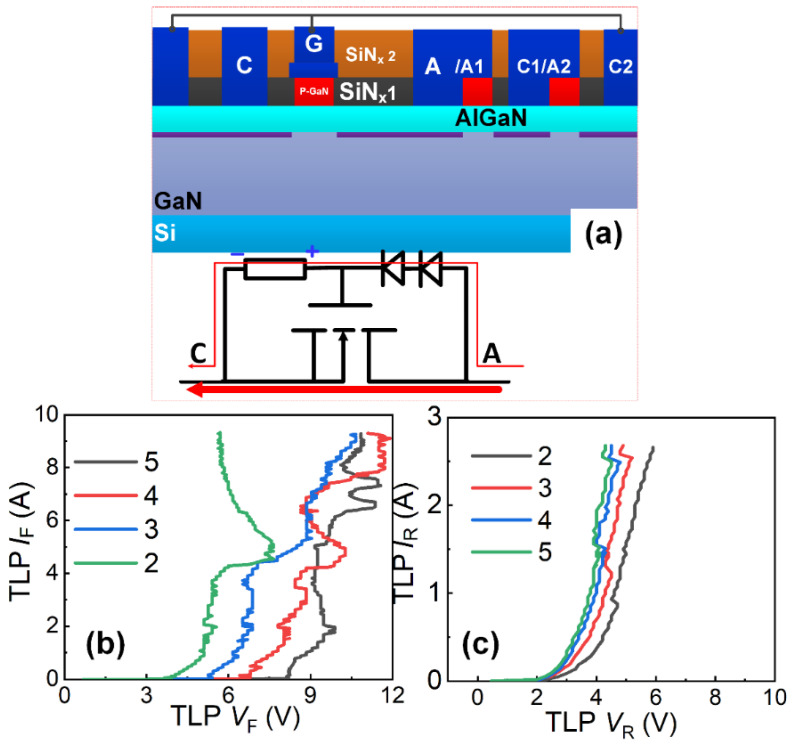
(**a**) Schematic diagram of the diode voltage-division unidirectional ESD protection structure and its forward conduction model; the influence of the number of diodes on (**b**) Forward TLP I-V characteristics and (**c**) Reverse TLP I-V characteristics of the diode voltage-division unidirectional ESD protection structure [13,14,20].

Figure 9a illustrates the resistive voltage-division unidirectional ESD protection structure, comprising a triggering P-GaN HEMT, a low-side resistor *R*_1_, and a high-side resistor *R*_2_. The low-side resistor *R*_1_ connects the cathode terminal to the gate terminal of the triggering P-GaN HEMT, while the high-side resistor *R*_2_ links the anode terminal to the gate terminal. Figure 10a depicts the diode voltage-division unidirectional ESD protection structure, consisting of a triggering P-GaN HEMT, low-side resistor *R*_1_, and a diode string. The low-side resistor *R*_1_ connects to the cathode terminal and the triggering P-GaN HEMT’s gate terminal, with the diode string attached between the anode terminal and gate terminal. Both the resistive voltage-division and diode voltage-division unidirectional ESD protection structures can be monolithically integrated into the power P-GaN HEMT: the protection structure’s anode terminal connects to the power P-GaN HEMT’s gate terminal, and the cathode terminal connects to the power device’s source terminal. Leveraging manufacturing processes fully compatible with the power P-GaN HEMTs, both the resistive voltage-division and diode voltage-division unidirectional ESD protection structure enable zero-process-modification integration.

#### 4.1.2. Operating Principle and Characteristics of Resistive Voltage-Division Structure [13,14,20]

During the forward ESD stress events (the anode-to-cathode ESD stress events), the electrostatic charges induced by the anode-to-cathode ESD stress will generate transient high voltage at the anode terminal of the resistive voltage-division unidirectional ESD protection structure. This transient voltage is divided by the voltage division path (high-side resistor *R*_2_ and low-side resistor *R*_1_). When the triggering P-GaN HEMT’s gate voltage reaches its forward conduction threshold voltage *V*_TH_F_, a 2DEG channel forms beneath the gate region of the triggering P-GaN HEMT. Consequently, the transient charges induced by the anode-to-cathode ESD stress can be rapidly discharged through the triggering P-GaN HEMT (Figure 9a). As the gate leakage current is negligible compared to the current *I*_R1_ through the low-side resistor *R*_1_ when the forward conduction threshold voltage *V*_TH_F_ is reached, the current *I*_R2_ through the high-side resistor *R*_2_ equals the current *I*_R1_ through the low-side resistor *R*_1_. Thus, the forward triggering voltage *V*_trig_F_ of this unidirectional ESD protection structure is proportional to the forward conduction threshold voltage of the triggering P-GaN HEMT with a ratio of (*R*_2_ + *R*_1_)/*R*_1_. Adjusting high-side resistor *R*_2_ and low-side resistor *R*_1_ enables an ideal forward triggering voltage for the resistive voltage-division unidirectional ESD protection structure (Figure 9b). Under the reverse ESD stress events (the cathode-to-anode ESD stress events), the triggering P-GaN HEMT activates when the cathode’s transient voltage exceeds the reverse triggering voltage *V*_trig_R_ (*V*_trig_R_ = *V*_TH_Rev_, where *V*_TH_Rev_ is the reverse conduction threshold voltage). The transient charges from the reverse ESD stress are then rapidly discharged through the triggering P-GaN HEMT (Figure 9a). Figure 9b presents the bidirectional TLP I-V characteristics of the resistive voltage-division unidirectional ESD protection structure. Taking *R*_2_/*R*_1_ of 10 kΩ/3 kΩ as example, the unidirectional ESD protection structure demonstrates a low triggering voltage (less than 10 V/3 V at the anode-to-cathode/cathode-to-anode ESD stress events) and high breakdown current (over than 9 A), demonstrating effective electrostatic charge dissipation and voltage clamping during both forward/reverse transient ESD events, thereby preventing ESD damage and enhancing the protected device robustness. However, during the reverse ESD events, the resistive voltage-division unidirectional ESD protection structure operates in diode mode with an undesirably low reverse triggering voltage (Figure 9b). Furthermore, the voltage division path (high-side resistor *R*_2_ and low-side resistor *R*_1_) will introduce a substantial forward/reverse steady-state leakage current. And under reverse bias voltages, the diode-mode operation also exhibits a low turn-on voltage, subsequently causing significant leakage.

#### 4.1.3. Operating Principle and Characteristics of Diode Voltage-Division Structure [12,16]

Replacing the high-side resistor *R*_2_ in the resistive voltage-division unidirectional ESD protection structure with a diode string forms the diode voltage-division unidirectional ESD protection structure, effectively eliminating reverse leakage in the voltage division path. During the forward ESD stress events (the anode-to-cathode ESD stress events), the transient high voltage is divided by the diode string and low-side resistor *R*_1_. When the triggering P-GaN HEMT gate voltage reaches the forward conduction threshold voltage *V*_TH_F_, a 2DEG channel forms beneath the gate region of the triggering P-GaN HEMT, enabling rapid discharge of transient electrostatic charges. The forward triggering voltage *V*_trig_F_ is proportional to the forward conduction threshold voltage *V*_TH_F_ of the triggering P-GaN HEMT and depends on both the number of series diodes and low-side resistor *R*_1_. Adjusting low-side resistor *R*_1_ and diode count can achieve an optimal forward triggering voltage for the diode voltage-division unidirectional ESD protection structure (Figure 10b). Under the reverse ESD stress events (the cathode-to-anode ESD stress events), the triggering P-GaN HEMT activates when the cathode transient voltage exceeds the reverse triggering voltage *V*_trig_R_ (*V*_trig_R_ = *V*_TH_Rev_, the reverse conduction threshold voltage), facilitating rapid charge dissipation. Bidirectional TLP I-V characteristics of the diode voltage-division unidirectional ESD protection structure are shown in Figure 10b,c. Taking diode count of 5 as example, the unidirectional ESD protection structure demonstrates a low triggering voltage (less than 10 V/3 V at the anode-to-cathode/cathode-to-anode ESD stress events) and high breakdown current (over than 9 A), demonstrating effective electrostatic charge discharge and voltage clamping during transient events, preventing ESD damage and enhancing device robustness. However, during reverse ESD stress events, the diode voltage-division unidirectional ESD protection structure operates in diode mode with a low reverse triggering voltage (Figure 10c). Additionally, the voltage division path introduces substantial reverse steady-state leakage currents. Under reverse DC bias, the diode-mode operation exhibits low turn-on voltage, subsequently causing significant leakage.

#### 4.1.4. Transient Charge Modulation-Based Unidirectional ESD Protection [15]

**Figure 11:**
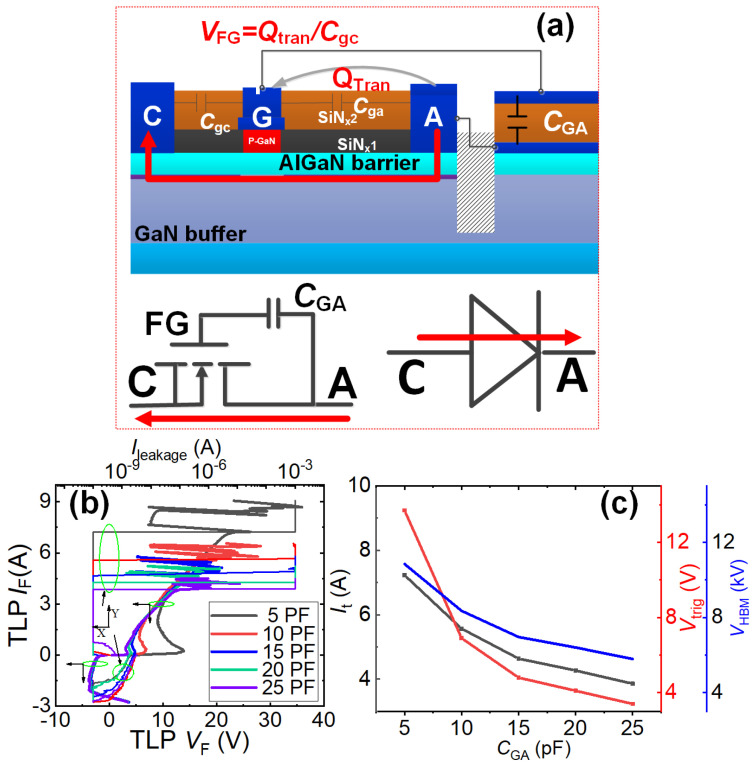
(**a**) Unidirectional ESD protection structure based on the transient charge modulation mechanism and its bidirectional conduction model; (**b**) Bidirectional TLP I-V curve; (**c**) Effect of the modulation capacitor on key characteristics [15].

Figure 11a illustrates the simplified schematic diagram and operating mechanism of the transient charge modulation-based unidirectional ESD protection structure, which resembles a conventional P-GaN HEMT but incorporates an additional pF-level capacitor (*C*_GA_) connected between the anode terminal and floating gate. Consequently, the transient charge modulation-based unidirectional ESD protection structure can be fabricated on the standard power P-GaN HEMT process platforms and seamlessly integrated with existing GaN power devices. As shown in Figure 11a, during the forward ESD stress events (the anode-to-cathode ESD stress events), the rapidly changing voltage at the anode (A) terminal can generate capacitive coupling current flowing from the anode (A) terminal to the floating gate region, then toward the cathode (C) terminal. The current delivers some transient charge (*Q*_tran_) to the floating gate region, lowering the energy band beneath the floating gate region. When the transient charge raises the potential of the floating gate beyond its threshold voltage, a 2DEG channel forms beneath the floating gate region, enabling rapid discharge of ESD-induced transient electrostatic charges.

Figure 11b presents the bidirectional TLP I-V characteristics for the transient charge modulation-based unidirectional ESD protection structure. Taking *C*_GA_ of 10 pF as example, the unidirectional ESD protection structure demonstrates a low triggering voltage (less than 10 V/3 V at the anode-to-cathode/cathode-to-anode ESD stress events) and high breakdown current (*I*_t_ = 5.6 A). This confirms effective dissipation of ESD-induced transient charges and voltage clamping, thereby enhancing the protected device’s ESD reliability and preventing damage. Figure 11c shows the impact of pF-level *C*_GA_ on the forward triggering voltage *V*_trig_F_, breakdown current *I*_t_, and equivalent HBM failure voltage *V*_HBM_ (calculated as *I*_t_ × 1500 Ω). Increasing *C*_GA_ from 5 pF to 25 pF reduces the forward triggering voltage *V*_trig_F_ from 13.8 V to 3.4 V, enabling optimal triggering voltage adjustment. Similarly, the breakdown current *I*_t_ decreases from 7.23 A to 3.86 A, resulting in a corresponding equivalent HBM failure voltage *V*_HBM_ reduction from 10.8 kV to 5.8 kV. While *C*_GA_ tuning optimizes the forward triggering voltage *V*_trig_F_, it compromises protection capability. Crucially, even at 25 pF, the unidirectional ESD protection structure’s equivalent HBM failure voltage *V*_HBM_ meets the industry benchmark (2 kV). Thus, this technology effectively prevents ESD damage while enhancing reliability. The absence of voltage division paths eliminates significant steady-state leakage. However, during reverse ESD events, the unidirectional ESD protection structure operates in diode mode with low reverse triggering voltage (Figure 11b).

### 4.2. Bidirectional ESD Protection Technology

In some application fields of power devices, it is necessary to apply a negative gate voltage to ensure the complete turn-off of the device gate channel and suppress false triggering. Therefore, the ESD protection technology with bidirectional clamping capability is required to clamp the gate potential to the desired voltage value during both forward and reverse ESD stress events while avoiding affecting the normal operation of the device. The following are several existing bidirectional ESD protection technologies based on the P-GaN HEMT process platform, all designed based on monolithic bidirectional switches: resistor voltage-division bidirectional ESD protection technology, diode voltage-division bidirectional ESD protection technology, bidirectional ESD protection structure based on common-drain bidirectional switches and transient charge modulation mechanism, and bidirectional ESD protection structure based on common-source bidirectional switches and transient charge modulation mechanism.

#### 4.2.1. Resistive Voltage-Division Bidirectional ESD Protection Technology [20]

**Figure 12:**
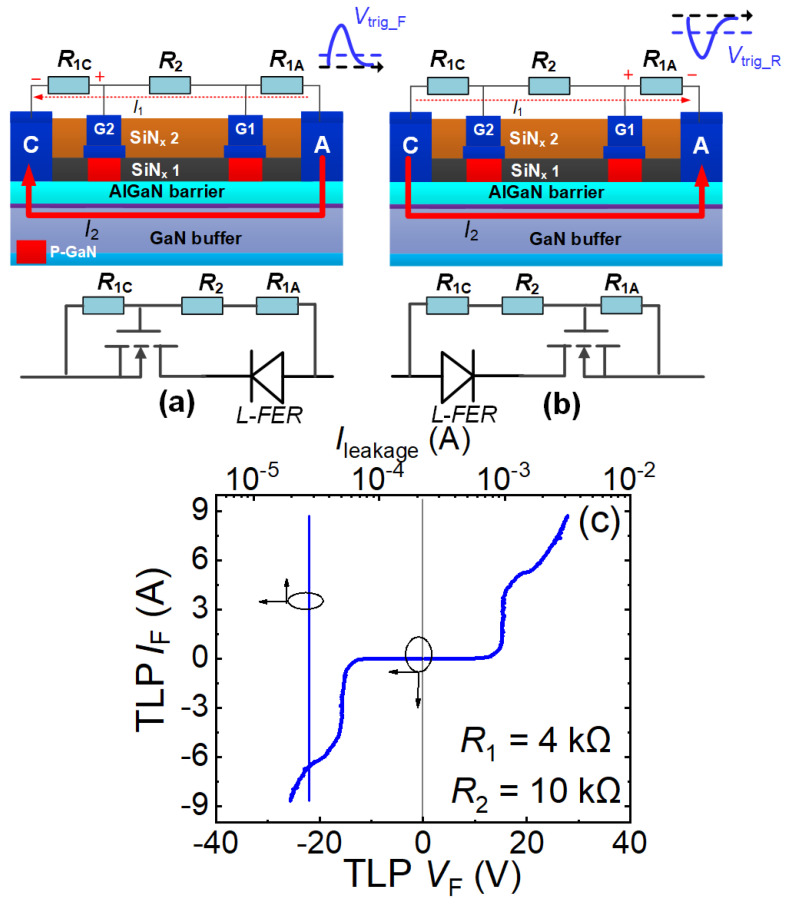
Resistive voltage-division bidirectional ESD protection structure: (**a**) Operational mechanism during forward ESD stress, (**b**) Operational mechanism during reverse ESD stress, (**c**) Bidirectional TLP I-V characteristics [20].

Figure 12a,b show the simplified schematic diagram of the resistive voltage-division bidirectional ESD protection structure and its bidirectional working mechanism. This bidirectional ESD protection structure consists of a small-sized monolithic bidirectional switch (MBS), two current-limiting resistors (*R*_1C_/*R*_1A_), and a scaling resistor (*R*_2_). Here, the current-limiting resistors *R*_1C_/*R*_1A_ are connected in parallel between the anode/cathode terminal and their adjacent gate, respectively, and the scaling resistor *R*_2_ is connected in parallel between the two gates. Similarly to the resistive voltage-division unidirectional ESD protection structure, the manufacturing process of the resistive voltage-division bidirectional ESD protection structure is completely identical to that of power P-GaN HEMTs, meaning this bidirectional ESD protection structure is very convenient to integrate with power P-GaN HEMTs.

The operational mechanism of the resistive voltage-division bidirectional ESD protection structure is illustrated in Figure 12a,b. During both forward and reverse ESD stress events (the anode-to-cathode/cathode-to-anode ESD stress events), this bidirectional structure functions as a combination of unidirectional ESD protection and a lateral field-controlled rectifier (L-FER). In forward ESD stress events (the anode-to-cathode ESD stress events), the first gate structure and anode terminal of the bidirectional protection serve as the L-FER, while the anode terminal, second gate structure, cathode terminal, the current-limiting resistors *R*_1C_/*R*_1A_, and the scaling resistor *R*_2_ constitute the unidirectional ESD protection component (Figure 12a). The L-FER activates at extremely low voltages. The transient voltages induced by the electrostatic charges generate forward current through the current-limiting resistors *R*_1C_/*R*_1A_ and the scaling resistor *R*_2_, creating a transient voltage drop between the second gate and cathode terminal. When this voltage drop exceeds the threshold voltage of the second gate structure in the bidirectional switch, the 2DEG channel beneath it activates, enabling rapid discharge of transient electrostatic charges from the forward ESD stress events (the anode-to-cathode ESD stress events). Adjusting the current-limiting resistors *R*_1C_/*R*_1A_ and the scaling resistor *R*_2_ allows optimization of the forward triggering voltage *V*_trig_F_. Similarly, modifying these resistors achieves an ideal reverse triggering voltage. During reverse ESD stress events (the cathode-to-anode ESD stress events), the second gate structure and cathode terminal operate as the L-FER, while the cathode terminal, first gate structure, anode terminal, the current-limiting resistors *R*_1C_/*R*_1A_, and the scaling resistor *R*_2_ form the unidirectional protection component (Figure 12b). The transient voltages induce a reverse current through the resistors, causing voltage drop between the first gate and anode terminal. When this drop surpasses the threshold voltage of the first gate structure, the transient charges from the reverse ESD stress events (the cathode-to-anode ESD stress events) can be rapidly discharged.

Bidirectional TLP testing (Figure 12c) revealed that with *R*_1C_/*R*_1A_ = 4 kΩ and *R*_2_ = 10 kΩ, the resistive voltage-division bidirectional ESD protection structure exhibits a forward triggering voltage *V*_trig_F_ of 12.69 V and a reverse triggering voltage *V*_trig_R_ of −12.9 V, demonstrating symmetrical clamping capability. With breakdown currents exceeding 8 A, it effectively dissipates transient electrostatic charges during ESD stress events, clamping protected device potentials at low levels and significantly enhancing ESD robustness. However, the voltage division path (the current-limiting resistors *R*_1C_/*R*_1A_ and the scaling resistor *R*_2_) will introduce substantial forward and reverse steady-state leakage currents.

#### 4.2.2. Diode Voltage-Division Bidirectional ESD Protection Technology [21]

**Figure 13:**
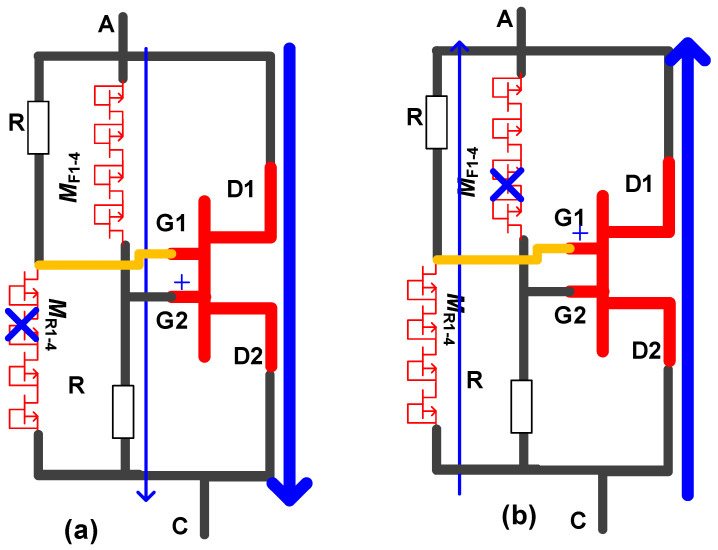
Operational mechanism of the diode voltage-division bidirectional ESD protection structure during (**a**) forward and (**b**) reverse operation [21].

The circuit schematic of the diode voltage-division bidirectional ESD protection structure is shown in Figure 13a, comprising a forward trigger circuit, a reverse trigger circuit, and a bidirectional switch serving as the discharge path. The structural schematic of the bidirectional switch is same as that in Figure 12a, where its two gates are controlled by the forward and reverse trigger circuits, respectively. The forward trigger circuit consists of a forward rectifier diode chain (*M*_F1-4_) and a resistor (*R*), while the reverse trigger circuit is identical to the forward trigger circuit.

During the forward ESD stress events (the anode-to-cathode ESD stress events), the transient electrostatic charge induced by the ESD stress events will introduce a large transient voltage drop across the forward trigger circuit. The forward rectifier diode chain (*M*_F1-4_) turns on and withstands part of the transient voltage. Simultaneously, the remaining portion of the transient voltage is applied between the first gate (G1) terminal of the bidirectional switch and the first ohmic contact (S1, connected to the cathode terminal) (*V*_G1S1_), causing the 2DEG channel under the first gate terminal G1 to conduct. Meanwhile, the reverse rectifier diode chain *M*_R1-4_ is turned off and withstands most of the transient voltage, resulting in a very small voltage between the second gate (G2) terminal of the bidirectional switch and the second ohmic contact (S2, connected to the anode terminal) (*V*_G2S2_). In this way, the discharge bidirectional switch can operate as a series-connected forward-biased diode and resistor (Figure 13a), allowing the transient electrostatic charge induced by the forward ESD stress events to be rapidly discharged through the bidirectional switch. The transient electrostatic charge induced by the reverse ESD stress events (the cathode-to-anode ESD stress events) can be discharged rapidly in the same manner, as shown in Figure 13b. So, the diode voltage-division bidirectional ESD protection structure can effectively protect the device against ESD damage and enhance its ESD robustness. However, the forward/reverse trigger circuits of the bidirectional ESD protection structure also introduce relatively large forward/reverse static leakage currents.

#### 4.2.3. Transient Charge Modulation-Based Bidirectional ESD Protection [18,19]

◆ With Common-Drain Bidirectional Switch as Discharge Path

**Figure 14:**
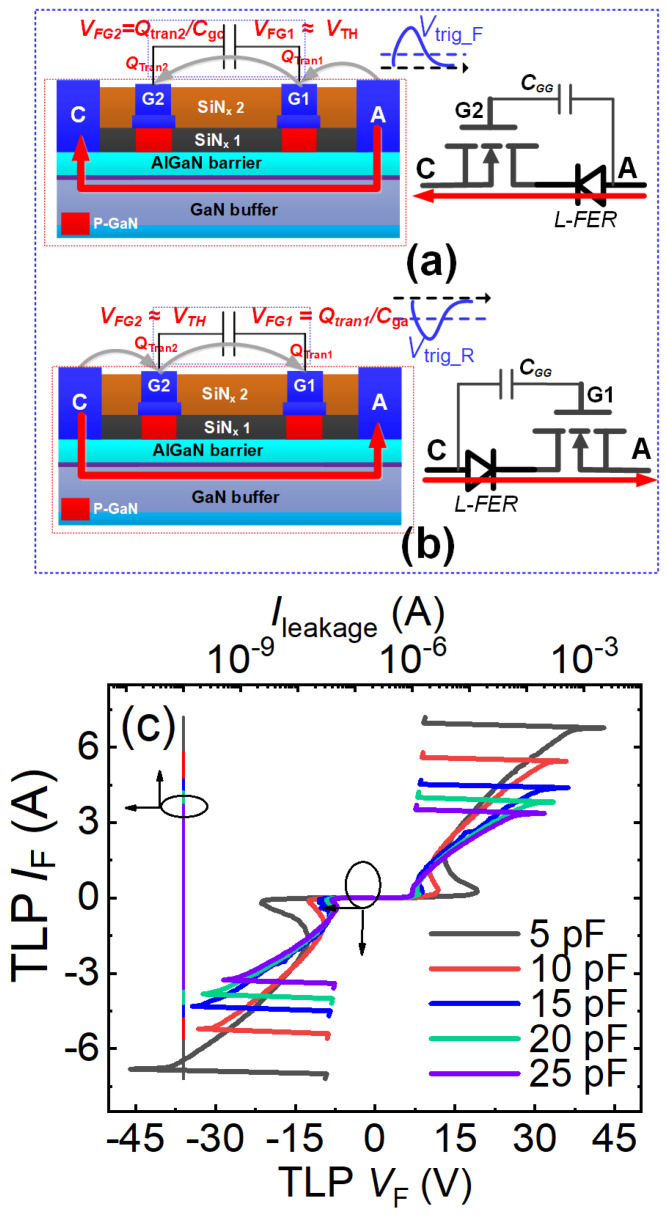
Bidirectional ESD protection structure based on the transient charge modulation mechanism: Operational mechanism of the bidirectional ESD protection structure using a common-drain bidirectional switch as the discharge path: (**a**) During forward ESD stress, (**b**) During reverse ESD stress, (**c**) Bidirectional TLP I-V characteristics [18].

Figure 14a,b illustrate the bidirectional ESD protection structure based on a common-drain bidirectional switch and transient charge modulation mechanism. This configuration features a floating-gate common-drain bidirectional switch and a regulating capacitor (*C*_GG_) connected between the two floating gates. Mirroring the transient charge modulation-based unidirectional ESD protection structure, this bidirectional structure can be fabricated on conventional power P-GaN HEMT process platforms and seamlessly integrated with existing GaN power devices, enabling streamlined ESD protection design for power P-GaN HEMTs. The bidirectional operation mechanism is shown in Figure 14 a,b: During forward ESD stress events (the anode-to-cathode ESD stress events) (Figure 14a), the rapidly changing transient voltage at the anode terminal generates capacitive coupling current flowing from the anode terminal through both floating gates to the cathode terminal. The current delivers transient charge (*Q*_tran_) to the floating gate region, lowering the energy band beneath it. When both floating gate potentials exceed their threshold voltages, the 2DEG channels beneath them activate, enabling rapid discharge of forward ESD-induced transient charges. During the reverse ESD stress events (the cathode-to-anode ESD stress events) (Figure 14b), the rapidly changing transient voltage at the cathode terminal similarly produces capacitive coupling current flowing from the cathode terminal through both floating gates to the anode terminal. Activation occurs when floating gate potentials surpass threshold voltages, facilitating discharge of the reverse ESD-induced transient charges.

In both forward and reverse ESD stress events, this bidirectional ESD protection structure can be regarded as a combination of a unidirectional ESD protection structure and a lateral field-controlled rectifier (L-FER). During the forward ESD stress events (the anode-to-cathode ESD stress events), the first gate structure and anode terminal of this bidirectional ESD protection structure function as the lateral field-controlled rectifier (L-FER), while its anode terminal, second gate structure, cathode terminal, and adjustable capacitor collectively constitute the unidirectional ESD protection structure. The voltage required to simultaneously turn on the 2DEG channels beneath both floating gates (the forward trigger voltage, *V*_trig_F_) is proportional to the threshold voltage and *C*_gc_/(*C*_gg_ + *C*_GG_), where *C*_gg_ represents the parasitic capacitance between the two floating gates and *C*_gc_ denotes the parasitic capacitance between the second gate structure and the cathode terminal. By adjusting the regulating capacitor *C*_GG_, the desired forward trigger voltage *V*_trig_F_ can be obtained. During the reverse ESD stress events (the cathode-to-anode ESD stress events), the second gate structure and cathode terminal of this ESD protection structure act as the lateral field-controlled rectifier (L-FER), while its cathode terminal, first gate structure, anode terminal, and adjustable capacitor collectively form the unidirectional ESD protection structure. In this case, the voltage required to simultaneously turn on the 2DEG channels beneath both floating gates (the reverse trigger voltage *V*_trig_R_) is proportional to the threshold voltage and *C*_ga_/(*C*_gg_ + *C*_GG_). Here, *C*_ga_ is the parasitic capacitance between the first gate structure and the anode. By modifying the regulating capacitor *C*_GG_, the required reverse trigger voltage *V*_trig_R_ can also be achieved.

Figure 14c presents the bidirectional TLP I-V characteristics of this bidirectional ESD protection structure based on the common-drain bidirectional switch and transient charge modulation mechanism. Taking *C*_GG_ = 10 pF as an example, the bidirectional ESD protection structure exhibits forward and reverse trigger voltages of 10 V and a bidirectional breakdown current as high as 5 A. This demonstrates its capability to rapidly discharge accumulated electrostatic charges and effectively clamp the voltage at critical nodes during both forward and reverse transient ESD events. Furthermore, the trigger voltage of this bidirectional ESD protection structure is higher than that of its unidirectional counterpart. This is because triggering the bidirectional structure requires simultaneously turning on the 2DEG channels beneath two gates. To achieve a lower trigger voltage, this can be realized by adjusting the value of the adjustable capacitor. As described, the adjustable capacitor plays a crucial role in modulating the bidirectional TLP I-V characteristics of this bidirectional ESD protection structure. Both the bidirectional trigger voltage and the breakdown current decrease as the adjustable capacitor increases, as shown in Figure 14c. When the capacitance of the adjustable capacitor increases from 5 pF to 25 pF, the bidirectional trigger voltage decreases from 19.7 V to 7.2 V, and the breakdown current drops from 6.77 A to 3.38 A. Correspondingly, the equivalent human body model failure voltage of this bidirectional ESD protection structure reduces from 10.2 kV to 5.07 kV. Additionally, since this ESD protection structure lacks additional voltage-division paths, it does not introduce significant steady-state leakage current.

◆ With Common-Source Bidirectional Switch as Discharge Path

**Figure 15:**
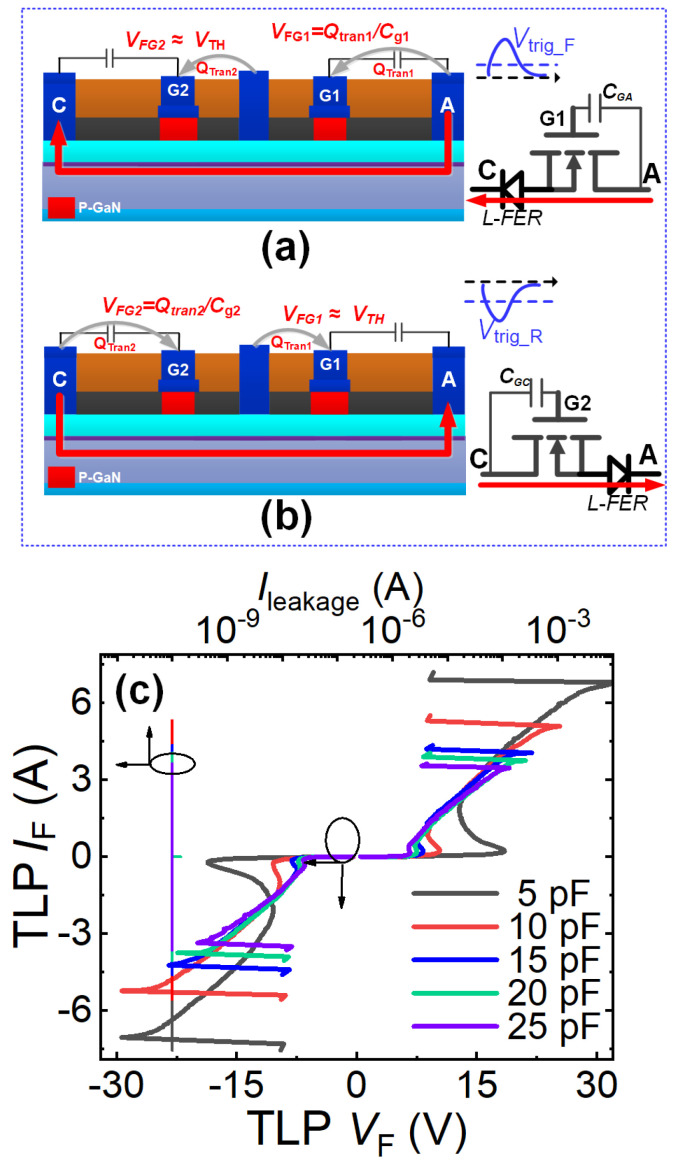
Bidirectional ESD protection structure based on the transient charge modulation mechanism: Operational mechanism of the bidirectional ESD protection structure using a common-source bidirectional switch as the discharge path: (**a**) During forward ESD stress, (**b**) During reverse ESD stress; (**c**) Bidirectional TLP I-V characteristics [19].

Figure 15a,b present the schematic diagram and operating mechanism of a bidirectional ESD protection structure based on a common-source bidirectional switch and transient charge modulation principles. This bidirectional ESD protection structure features a floating-gate common-source bidirectional switch and two tuning capacitors: specifically, it has two floating gate electrodes, a common-source ohmic contact between the floating gate electrodes, two ohmic contacts acting as anode/cathode electrodes, and two pF-level tuning capacitors (designated as *C*_GA_ and *C*_GC_) connected in parallel between the anode/cathode and their adjacent floating gate electrodes. It can be seen that this bidirectional ESD protection structure resembles two P-GaN HEMTs with their sources connected together. The operating mechanism under the forward ESD stress events (the anode-to-cathode ESD stress events) is illustrated in Figure 15a. During the forward ESD stress events (the anode-to-cathode ESD stress events), a rapidly changing transient voltage at the anode terminal can induce a capacitively coupled current flowing from the anode terminal to the cathode terminal. This capacitively coupled current carries a certain amount of transient charge to the two floating gate electrodes, which can lower the energy band in the floating gate region. When the gate potential induced by the transient charge exceeds the threshold voltage of the 2DEG channel, the 2DEG channel beneath the floating gate region will be turned on. Consequently, the large amount of transient charge caused by the forward ESD stress events can be rapidly discharged through the activated 2DEG channel beneath the floating gate region. The operating mechanism under the reverse ESD stress events (the cathode-to-anode ESD stress events), shown in Figure 15b, is consistent with that under the forward ESD stress events (the anode-to-cathode ESD stress events).

Figure 15c shows the bidirectional TLP I-V characteristics of this bidirectional ESD protection structure based on the common-source bidirectional switch and transient charge modulation mechanism. Using tuning capacitors of 5 pF as an example, the structure exhibits a bidirectional trigger voltage of 18 V and a bidirectional failure current of 7A, demonstrating its effectiveness in rapidly dissipating accumulated electrostatic charge and clamping critical node voltages during both forward and reverse transient ESD events. It can also be seen from Figure 15c that variations in *C*_GA_/*C*_GC_ significantly affect the bidirectional TLP I-V characteristics. The trigger voltage of this bidirectional ESD protection structure decreases as *C*_GA_/*C*_GC_ increases. Similarly, the failure current also reduces with an increase in *C*_GA_/*C*_GC_. Therefore, although adjusting the tuning capacitors can tune the trigger voltage, it also weakens the protection capability. Furthermore, as this ESD protection structure does not incorporate additional voltage-division paths, it does not introduce significant steady-state leakage current.

### 4.3. Ohm/Sch Hybrid (Hyb.) Gate Technology [27]

**Figure 16:**
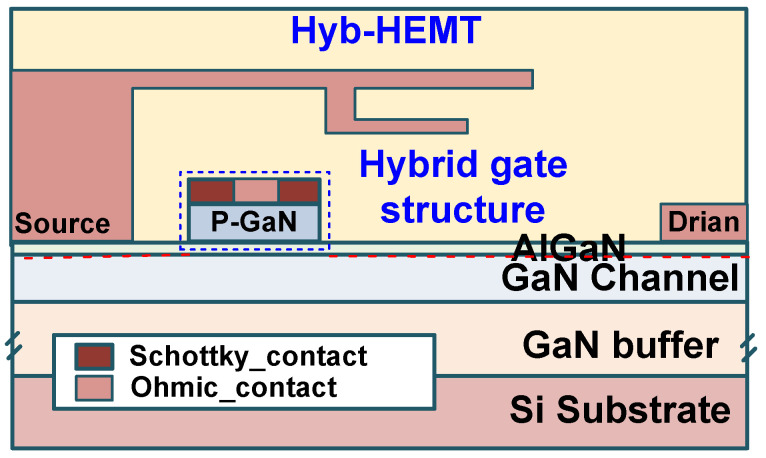
Cross-sectional schematic diagram of the HEMT with an Ohmic/Schottky hybrid (Hyb.) gate structure (Hyb-HEMT) [27].

The aforementioned ESD protection techniques all require additional integrated protection structures outside the power P-GaN HEMT, which inevitably increases chip area and cost. To address this, Ma et al. from Southeast University further proposed the Ohm/Sch Hybrid (Hyb.) Gate Technology [27]. As previously mentioned, the ESD robustness of the ohmic-type P-GaN gate structure is superior to that of the Schottky-type P-GaN gate structure. By combining the ohmic-type and Schottky-type P-GaN gate structures, it is possible to achieve better gate ESD robustness than that of Schottky-gate P-GaN HEMTs, as shown in Figure 16. The hybrid gate structure consists of Schottky-type metal and ohmic-type metal, with the ohmic-type metal placed at the center of the Schottky-type region. The ohmic gate region is formed by depositing a Ti/Au metal stack using an electron beam evaporator. Then, W metal is deposited on top of the p-GaN layer and the ohmic region to form the Schottky contact. The breakdown current of the Schottky-gate P-GaN HEMT is less than 13.9 mA/mm [27]. In contrast, the breakdown current of the Hyb-HEMT is significantly improved to 153.7 mA/mm, indicating that the hybrid gate structure can greatly enhance the gate ESD tolerance of P-GaN HEMTs for forward ESD protection [27].

## 5. Prospect

The primary advantage of the ESD protection technologies described in this work, based on the P-GaN HEMT platform, lies in the full compatibility between the fabrication process of these ESD protection structures and that of the protected P-GaN HEMTs. This enables the monolithic integration of the ESD protection structures with the protected P-GaN HEMTs. Such integration not only effectively enhances the ESD reliability of the gate structure of the protected P-GaN HEMTs but also significantly reduces the fabrication cost of the P-GaN HEMT chips. Furthermore, it helps minimize the parasitic parameters of GaN-based power systems, thereby improving overall system performance.

**Figure 17:**
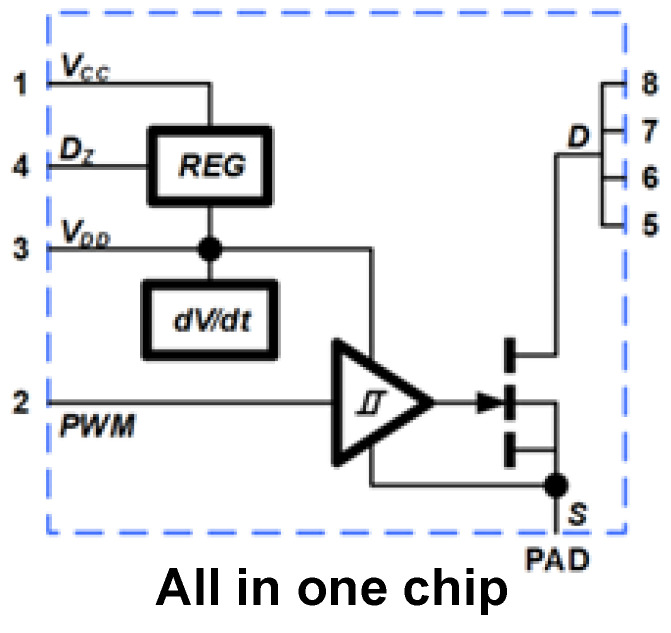
The on-chip monolithic integration protection technologies (NV6115) [48].

**Figure 18:**
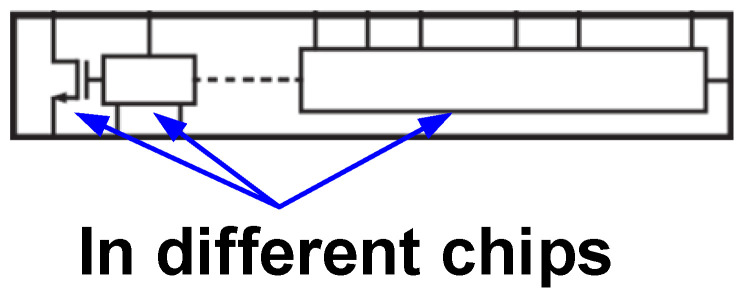
The package-level multi-chip integration protection technologies (InnoSwitch4-QR) [49].

Current integrated ESD protection technologies and similar safeguarding approaches can be primarily categorized into three types: on-chip integration protection [28,29,30,31,32,33,34,35,36], package-level protection [37,38,39,40,41], and system-level protection [42,43,44,45,46,47]. For on-chip monolithic integration protection (representative GaN product is as shown in Figure 17), the core technology involves utilizing the mature P-GaN HEMT process platform to monolithically integrate ESD protection structures with the protected P-GaN HEMTs on the same chip through co-design and optimization. For instance, by fabricating both the GaN ESD protection structures mentioned in this work and the protected P-GaN HEMTs on the same chip, connecting the cathode of the GaN ESD protection structure to the source of the protected P-GaN HEMT and the anode to its gate, the GaN ESD protection structure can be placed as close as possible to the protected P-GaN HEMT. This minimizes system parasitic parameters to the greatest extent, enables high-speed transient response, and improves overall system efficiency. The package-level multi-chip integration protection scheme (representative GaN product is as shown in Figure 18) involves integrating dedicated ESD protection chips with the protected P-GaN HEMTs within a single package using techniques such as wire bonding or flip-chip bonding. While this approach circumvents process compatibility issues and provides robust protection, it inevitably introduces package parasitic inductance and resistance, which can limit response speed and potentially impact high-frequency performance. In contrast, system-level integrated ESD protection involves placing discrete ESD protection components at the circuit board level. This method offers design flexibility, but because the protection components are located far from the core chip, the parasitic effects along the protection path are most significant, severely affecting the system’s transient response capability.

Since P-GaN HEMT are planar devices, they can readily be monolithically integrated with other protection structures (such as ESD protection, overcurrent protection, and overvoltage protection). Moreover, on-chip monolithic integration protection offers two additional advantages over package-level multi-chip integration and system-level integration schemes. First, it provides the fastest response speed, as the protection structure is placed extremely close to the protected P-GaN HEMT without the extra parasitic parameters introduced by package interconnects. Second, when the fabrication process of the protection structure is fully compatible with that of the protected P-GaN HEMT, no additional fabrication steps are required to implement the protection structure, significantly reducing chip fabrication costs. Therefore, the on-chip monolithic integration ESD protection scheme is poised to become the mainstream direction for the future development of P-GaN HEMTs.

## 6. Conclusions

This paper summarizes the gate ESD failure behaviors of P-GaN HEMTs with different gate structures and reviews existing GaN-based ESD protection technologies. Research indicates that Schottky-gate P-GaN HEMTs lack effective electrostatic charge discharge paths, leading to transient charge accumulation at the gate under forward/reverse ESD stresses, which generates overvoltage and causes gate breakdown failure. Their equivalent Human Body Model (HBM) failure voltages fall below industry standards. To enhance gate ESD protection, existing technologies are primarily classified into unidirectional and bidirectional schemes. Unidirectional ESD protection techniques—including resistive voltage-division, diode voltage-division, and transient charge modulation approaches—can discharge forward/reverse transient charges but exhibit significant limitations: they operate in diode mode during reverse ESD events resulting in excessively low reverse triggering voltages; resistive/diode voltage-division structures introduce large forward static gate leakage currents; while transient charge modulation allows flexible triggering voltage adjustment via capacitors, increasing capacitance reduces breakdown current and equivalent HBM failure voltage, thereby compromising protection capability. For applications requiring negative gate bias, bidirectional ESD protection technologies—encompassing resistive/diode voltage-division and transient charge modulation with common-drain/common-source bidirectional switches—achieve balanced potential clamping through symmetric designs or bidirectional switches. These ensure stable gate potential limitation at set values during bidirectional ESD events, effectively discharge transient charges, significantly enhance the ESD robustness of protected devices, and facilitate monolithic integration with GaN power devices. Nevertheless, voltage-division bidirectional structures still introduce substantial static leakage currents, and increasing modulation capacitance in transient charge modulation technologies also reduces breakdown current and protection capability.

## Data Availability

No new data were created or analyzed in this study.
